# Bloodstream infections caused by multidrug-resistant gram-negative bacteria: epidemiological, clinical and microbiological features

**DOI:** 10.1186/s12879-019-4265-z

**Published:** 2019-07-11

**Authors:** Helena Ferreira Leal, Jailton Azevedo, Giulyana Evelyn Oliveira Silva, Angelica Maria Lima Amorim, Larissa Rangel Cabral de Roma, Ana Carolina Palmeira Arraes, Edilane Lins Gouveia, Mitermayer Galvão Reis, Ana Verena Mendes, Marcio de Oliveira Silva, Maria Goreth Barberino, Ianick Souto Martins, Joice Neves Reis

**Affiliations:** 1 Laboratory of Pathology and Molecular Biology (LPBM), Gonçalo Moniz Research Institute, Oswaldo Cruz Foundation, Candeal, Salvador, Bahia 40296-710 Brazil; 2 Laboratory of Research on Clinical Microbiology (LPMC), School of Pharmacy, Federal University of Bahia, Ondina, Salvador, Bahia 40170-115 Brazil; 3 São Rafael Hospital, São Marcos, Salvador, Bahia 41253-190 Brazil; 4 Bahia Hospital, Pituba, Salvador, Bahia 40280-000 Brazil; 5 Bahiana School of Medicine and Public Health of the Bahia Foundation for the Development of Sciences, Salvador, Bahia Brazil; 6 Faculty of Medicine, Fluminense Federal University Downtown, 24033-900, Niterói, Rio de Janeiro, Brazil

**Keywords:** Bacteremia, Antibiotic resistance, Gram-negative bacteria

## Abstract

**Background:**

Bloodstream infections (BSI) are associated with high morbidity and mortality. This scenario worsens with the emergence of drug-resistant pathogens, resulting in infections which are difficult to treat or even untreatable with conventional antimicrobials. The aim of this study is to describe the epidemiological aspects of BSI caused by multiresistant gram-negative bacilli (MDR-GNB).

**Methods:**

We conducted a laboratory-based surveillance for gram-negative bacteremia over a 1-year period. The bacterial isolates were identified by MALDI-TOF/MS and the antimicrobial susceptibility testing was performed by VITEK®2. Resistance genes were identified through PCR assays.

**Results:**

Of the 143 patients, 28.7% had infections caused by MDR-GNB. The risk factors for MDR bacteremia were male sex, age ≥ 60, previous antimicrobial use, liver disease and bacteremia caused by *K. pneumoniae*. *K. pneumoniae* was the most frequently observed causative agent and had the highest resistance level. Regarding the resistance determinants, SHV, TEM, OXA-1-like and CTX-M-gp1 were predominant enzymatic variants, whereas CTX-M-gp9, CTX-M-gp2, KPC, VIM, GES, OXA-48-like, NDM and OXA-23-like were considered emerging enzymes.

**Conclusions:**

Here we demonstrate that clinically relevant antibiotic resistance genes are prevalent in this setting. We hope our findings support the development of intervention measures by policy makers and healthcare professionals to face antibiotic resistance.

**Electronic supplementary material:**

The online version of this article (10.1186/s12879-019-4265-z) contains supplementary material, which is available to authorized users.

## Background

Bloodstream infections (BSI) are characterized as severe disorders since they are acute events and usually result in serious life-threatening organ dysfunctions, such as sepsis and septic shock [1, 2]. Sepsis is considered to be a public health issue and is a leading cause of mortality worldwide, being recently listed as a global health priority by World Health Organization [3, 4]. For dynamic infections such as BSI, the accurate diagnosis and administration of appropriate antimicrobial therapy are essential to enhance a patient’s survivability, thereby reducing the high frequencies of morbidity and mortality [5–8].

Although the development of antimicrobial resistance control guidelines by healthcare agencies represents important measures to ensure patient care and to implement antimicrobial stewardship strategies, these should be personalized according to the background of each geographic location. Therefore, it is necessary to understand local epidemiology through clinical and microbiological monitoring [9–11].

The epidemiology of BSI has been described extensively in many different countries [12–16]. However, when coming to the antibiotic resistance issue, only a few investigations conduct further research into detecting resistance genes [15, 17]. Likewise, many studies tackling the antibiotic resistance rely on the problem of screening resistant isolates solely, which may substantially affect susceptibility reports, representing a strong bias towards resistance [18].

In order to provide epidemiological and microbiological information about the occurrence of BSI due to gram-negative-bacilli (GNB), we conducted a prospective laboratory-based surveillance for BSI caused by GNB from 2015 through 2016 in tertiary referral hospital in the city of Salvador. Not only we expect that the evidence presented here give rise to intervention strategies for effective antimicrobial therapy, but also foster early measures to control the spread of MDR-GNB.

## Methods

### Study design and population

A prospective surveillance of patients with positive blood culture was conducted at the São Rafael Hospital (HSR) from March 2015 to March 2016. The HSR addresses medium and high complexity procedures. The population studied was composed of patients without age restriction with BSI due to GNB, defined by positive blood culture with signs and symptoms of infection. Epidemiological and clinical characteristics of patients with BSI caused by S-GNB and MDR-GNB were compared.

### Data collection and definitions

Demographic and clinical data was collected through a review of medical records. The clinical characteristics addressed included: hospitalization unit, hospitalization during the previous 6 months, previous healthcare assistance, previous infections, comorbidities, presented symptoms, possible sources and risk factors for bacteremia, use of antibiotics in the previous 6 months, empirical and culture-guided therapy and clinical outcome in 30 days. The Charlson comorbidity index was used to measure the severity of underlying conditions at the time of admission, and the severity of the BSI episode was measured using the Pitt bacteremia score [19, 20].

The location of acquisition and type of BSI were defined as follows: 1) “community-onset - healthcare-associated” (CO-HCA) was defined as a BSI occurring within 48 h of hospital admission plus the presence of one of the following healthcare risk factors: prior hospitalization, surgery, dialysis, or residence in a long-term care facility within the 12 months preceding the BSI, or the presence of an invasive device; 2) “community-acquired” (CA) was defined as a BSI occurring ≤48 h after admission but without one of the above healthcare risk factors; and 3) “hospital-onset-healthcare-associated” (HO-HCA) was defined as a BSI that occurred 48 h after hospital admission [12, 21].

The BSI episodes were also classified by their origin in primary and secondary infections. Primary BSI is laboratory-confirmed by positive blood culture, but it has no identifiable extravascular focus of infection. Secondary BSI is characterized by the occurrence of a positive blood culture or clinical signs of sepsis in the presence of infection elsewhere [22, 23].

The conditions of sepsis, severe sepsis and septic shock have been identified using definitions established by the 1991 Consensus Conference, which were updated in 2001 [24, 25]. The multidrug resistance phenotype was defined using the consensus definitions proposed by Magiorakos et al. [26]. Episodes of polymicrobial infections were those in which more than one species were found in the same blood culture.

### Microbial isolation and identification and antimicrobial susceptibility testing

The blood cultures were performed on BacT/ALERT® 3D (bioMeriéux-France) and microbial identification was performed by MALDI-TOF MS (bioMeriéux-France) as described by Barberino et al. [27]. All GNB identified were stored in Trypticase Soy Broth (TSB) enriched with 10% glycerol at − 70 °C until it was reactivated for molecular tests.

The antimicrobial susceptibility profile of the enterobacteria was determined using a VITEK 2® (bioMeriéux-France). For non-fermentative GNB, antibiograms were manually performed using the agar-diffusion technique. The tests and the interpretative criteria were performed according to Clinical and Laboratory Standard Institute recommendations [28].

### Detection of antimicrobial resistance encoding genes

DNA extraction was performed from bacterial colonies cultured in TSA (Tryptic Soy Agar) for 18 to 24 h at 36 °C. The colonies were resuspended in 100 μl of sterile MilliQ water and heated at 95 °C for a period of 10 min. Subsequently, the content was centrifuged at 12,000 rpm for 2 min. The supernatant containing the genetic material was transferred to a sterile tube and stored at − 20 °C until PCR was performed.

Four multiplex PCR reactions were performed using the primer sequence and protocol developed by Dallene et al. [29] for the *bla*_TEM_, *bla*_SHV_, and *bla*_OXA-1-like_ genes; the *bla*_CTX-M (group 1, 2, 9)_; the *bla*_GES_ and *bla*_OXA-48-like_; and *bla*_KPC_ and *bla*_VIM_. In addition, three simplex reactions were performed to detect the presence of *bla*_IMP_, *bla*_NDM_ and *bla*_OXA-23-like_ genes using the primer sequences and protocol proposed by van der Zwaluw et al. [30] (Additional file 1: Table S1). The positive controls were bacterial DNA extracts from reference strains (Additional file 2: Table S2). The PCR products were electrophoresed as described by the two mentioned authors.

### Data management and statistical analyses

The data was managed and analyzed using Epi Info version 3.5.1 (CDC, Atlanta, GA, USA). For the epidemiological analysis, duplicate cases (i.e. patients who had experienced two episodes of BSI within 30 days and patients with polymicrobial infections) were excluded, so the episodes were analyzed based on the first entry.

Fisher’s exact or chi-square tests were used to compare the differences between the proportions for dichotomous variables, and the odds ratio (OR) and 95% confidence interval (CIs) were calculated as measures of association. Statistical significance was defined as *P* < 0.05. A multivariate logistic regression analysis was performed to identify the independent risk factors for MDR-GNB bacteremia. The variables introduced into the model included those with a crude *P*-value of< 0.25, those that were biologically sound, and those found in previous studies of BSI and MDR infections. We used backward elimination of any confounding variable based on the likelihood ratio test, using a significance cutoff of 0.05.

## Results

During the study period, 143 patients with bacteremia due to GNB were identified. Most of the patients were male (88, 61.5%) and were 60 years or older (73, 51.0%). The characteristics of the case patients are shown in Table 1.

**Table 1:** Epidemiological, demographic and clinical characteristics of patients with bloodstream infections (BSI) caused by gram-negative bacteria in tertiary referral hospital in Salvador, Brazil (*n =* 143)

Characteristics	MDR *n* (%) *n = 41* (28.7)	Non-MDR *n* (%) *n = 102* (71.3)	*p-value*	*OR (95% CI)*
Demographic data				
Male sex	31 (75.6)	57 (55.9)	0.03	2.45 (1.09–5.52)
Age groups (years), median (1 qt–3 qt)	63 (51–72)	57 (38–69)	0.03^+^	–
0–15	0 (0.0)	6 (5.9)	0.12	–
16–30	4 (9.8)	11 (10.8)	0.56	0.89 (0.26–2.99)
31–59	11 (26.8)	38 (37.3)	0.23	0.61 (0.28–1.37)
≥60	26 (63.4)	47 (46.1)	0.06	2.02 (0.96–4.27)
Comorbidities				
Charlson score, median (1 qt–3 qt) (*n =* 134)*	2.5 (2–4)	2 (2–3)	0.10	–
≥2 (*n =* 134)	20 (50.0)	39 (41.5)	0.37	1.41 (0.67–2.97)
Congestive heart failure (*n =* 134)	3 (7.5)	5 (5.3)	0.45	1.44 (0.33–6.35)
Cerebrovascular disease (*n =* 134)	5 (12.5)	11 (11.7)	0.55	1.08 (0.35–3.33)
Liver disease	7 (17.5)	5 (5.3)	0.02	3.78 (1.12–12.73)
Kidney disease	6 (15.0)	21 (22.3)	0.33	0.61 (0.23–1.66)
History of malignancy	16 (40.0)	42 (44.7)	0.62	0.82 (0.39–1.76)
Metastatic disease	7 (17.5)	16 (17.0)	0.95	1.03 (0.39–2.75)
HIV/AIDS	1 (2.5)	0 (0.0)	0.12	–
Previous antimicrobial use (*n =* 80)	29 (70.7)	51 (50.0)	0.02	2.41 (1.11–5.25)
Prophylactic	8 (19.5)	21 (20.6)	0.88	0.93 (0.37–2.32)
Therapeutic	26 (63.4)	43 (42.2)	0.02	2.37 (1.12–5.02)
Clinical information				
Hospitalized in prior 6 months (*n =* 140)	26 (65.0)	54 (54.0)	0.24	1.58 (0.74–3.38)
Long hospital stay (> 14 days)	30 (73.2)	70 (68.6)	0.59	1.2 (0.55–2.79)
Users of Health Insurance	31 (75.6)	73 (71.6)	0.62	1.23 (0.54–2.94)
Type of BSI				
Primary	24 (58.5)	56 (54.9)	0.69	1.16 (0.56–2.41)
Secondary (*n =* 63)	17 (41.5)	46 (45.1)	0.69	1.16 (0.56–2.41)
Urinary tract (*n =* 29)	7 (41.2)	22 (45.8)	0.74	0.82 (0.27–2.53)
Respiratory tract (*n =* 17)	5 (29.4)	12 (25.0)	0.72	1.25 (0.37–4.28)
Others (*n =* 19)	5 (29.4)	14 (29.2)	0.98	1.02 (0.30–3.41)
Location of acquisition				
Community-associated (*n =* 6)	1 (2.4)	5 (4.9)	0.45	0.48 (0.05–4.28)
Community-onset, healthcare associated (*n =* 44)	11 (26.8)	33 (32.4)	0.51	0.76 (0.34–1.71)
Hospital-onset, healthcare associated (*n =* 93)	29 (70.7)	64 (62.7)	0.36	1.43 (0.65–3.14)
Organisms (*n =* 170)**				
*Escherichia coli (n = 50)*	12 (24.5)	38 (31.4)	0.37	0.70 (0.33–1.50)
*Klebsiella pneumoniae (n = 50)*	27 (55.1)	23 (19.0)	<0.001	5.23 (2.53–0.77)
*Serratia marcescens (n = 15)*	1 (2.0)	14 (11.6)	0.04	0.15 (0.02–1.24)
*Enterobacter cloacae (n = 13)*	3 (6.1)	10 (8.3)	0.45	0.72 (0.19–2.75)
*Pseudomonas aeruginosa (n = 12)*	2 (4.1)	10 (8.3)	0.28	0.47 (0.09–2.24)
*Acinetobacter baumannii (n = 6)*	4 (8.2)	2 (1.7)	0.06	5.29 (0.93–9.88)
Others*** (*n =* 24)	0 (0)	24 (19.8)	<0.001	–
Polymicrobial BSI (*n =* 22)^Ω^	9 (18.4)	13 (10.7)	0.18	1.87 (0.74–4.71)

(^+^) Mann-Whitney U Test; (−) Not calculated; (*) Of the 143 patients, 134 had comorbidities according to their Charlson’s score. Regarding diabetes, diabetes with any organ demage, myocardial infarction, peripheral vascular disease, dementia, chronic obstructive pulmonary disease, connective tissue disease, peptic ulcer, hemiplegia or paraplegia and HIV/AIDS data are not shown since the number of patients with those comorbidities were limited (*n* < 5). None of the mentioned illnesses were risk or protective factor for MDR infection; (**) considering all isolates recovered; (***) *Aeromonas hydrophila* (*n =* 4), *Burkholderia cepacia* (*n =* 1), *Citrobacter koseri* (*n =* 1), *Elizabethkingia meningoseptica* (*n =* 1), *Enterobacter aerogenes* (*n =* 2), *Klebsiella oxytoca* (*n =* 1), *Moraxella osloensis* (*n =* 1), *Proteus mirabilis* (*n =* 4), *Pseudomonas putida* (*n =* 1), *Salmonella* spp.(*n =* 1), *Sphingomonas paucimobilis* (*n =* 1), and *Stenotrophomonas malthophilia* (*n =* 6); (Ω) in the study period 11 polymicrobial episodes were identified, and they involved 22 microbial isolates

Regarding the characteristics of the BSI episodes, most infections were primary 80/143 (55.9%). Among the secondary cases, the urinary tract (20.3%) was the most frequent site of origin, followed by the respiratory tract (11.8%). Concerning the location of acquisition, 93 (65.0%) episodes were HO-HCA, 44 (30.8%) were CO-HCA, and 6 (4.2%) were CA. The original site of the infection and the location of acquisition were not associated with the presence of the MDR pathogen.

Among the patients with CO-HCA infections (*n* = 44), the healthcare risk factors were a previous admission (*n =* 32), hemodialysis (*n =* 8), previous invasive procedure (*n =* 6), chemotherapy (*n =* 5) and homecare (*n =* 6). Among the patients with HO-HCA infections, the hospital settings in which they were allocated were hospital wards (*n =* 50), followed by the intensive care unit (*n =* 25), semi intensive care unit (*n =* 13) and emergency department (*n =* 2).

### Risk factors for MDR bacteremia

Among 143 episodes of BSI, 41 (28.7%) were caused by MDR-GNB. Univariate analysis performed to investigate the possible risk factors associated with BSI caused by MDR-GNB is presented in Table 1 and Table 2. Regarding the comorbidities addressed by the Charlson score, liver disease was positively associated with the presence of MDR pathogens. In addition, male sex, age ≥ 60 and the previous use of antimicrobial showed positive associations also.

**Table 2:** Clinical and prognostic characteristics of patients with bloodstream infections (BSI) caused by gram-negative bacteria in tertiary referral hospital in Salvador, Brazil (*n =* 143)

Characteristics	MDR *n* (%) *n = 41* (28.7)	Non-MDR *n* (%) *n = 102* (71.3)	*p-value*	*OR (95% CI)*
Potential risk factors				
Catheter use (*n =* 58)	15 (51.7)	43 (71.7)	0.06	0.42 (0.16–1.06)
Ventilator (*n =* 21)	5 (17.2)	16 (26.7)	0.32	0.57 (0.18–1.75)
Severity of illness				
Pitt score, median (1 qt–3 qt)	5 (1–10)	2 (0–6)	0.07^a^	–
0	8 (21.6)	33 (31.1)	0.27	0.61 (0.25–1.48)
1	6 (40.0)	35 (27.3)	0.31	1.77 (0.59–5.34)
2–3	4 (14.3)	37 (32.2)	0.06	0.35 (0.11–1.09)
≥4	23 (36.5)	18 (22.5)	0.06	1.9 (0.95–4.12)
Severe sepsis or septic shock^b^ (*n =* 76)	27 (65.9)	49 (48.0)	0.06	2.09 (0.99–4.43)
Crude mortality	23 (56.1)	27 (26.5)	<0.001	3.5 (1.65–7.62)

(^a^) Mann-Whitney U Test; (^b^) Sepsis complicated by organ dysfunction is termed severe sepsis, which could progress to septic shock, which is defined as “sepsis-induced hypotension persisting despite adequate fluid resuscitation”

Upon stratifying the previous use of antimicrobials in prophylactic and therapeutic, the statistical significance solely remained for the therapeutic use. Regarding the class of drugs used therapeutically, we found the use of fluoroquinolones and folate pathway inhibitors were more frequent in the MDR group (6, 23.1% vs. 4, 9.3% in the non-MDR group). However, this difference was not statistically significant (*P* = 0.24).

Concerning the etiology, *K. pneumoniae* infections had a positive association with the MDR phenotype (*P* < 0.05). Although *Acinetobacter baumannii* was more frequent in the MDR group than in the non-MDR group (8.2% vs. 1.7%), the statistical significance was not reached (*P* = 0.06). The presence of infections caused by *Serratia marcescens*, as well as the infections caused by the less frequently isolated bacteria (which were categorized into the “others” group), were negatively associated with the MDR phenotype.

Infections caused by MDR pathogens culminated in BSIs with high severity (Table 2). This association can be verified through the Pitt score, which was higher in patients with MDR infections (median 5 vs. median 2, *P =* 0.07) as well as the number of patients who experienced systemic complications due to bacteremia, such as severe sepsis and septic shock (65.9% vs. 48.0%). These indicators of severity were more frequent among MDR cases; however, it is important to clarify that none of these associations reached statistical significance. Infections caused by MDR pathogens were associated with death (*P* < 0.001) (Table 2).

A multivariate logistic regression was used to identify the factors that were independently associated with MDR infections. The results are shown in Table 3.

**Table 3:** Adjusted odds ratio for independent risk factors for multidrug resistant gram-negative bacteremia based on multivariate logistic regression analysis^a^

Risk factors	Adjusted multivariate	
	*p-value*	*OR (95% CI)*
Male sex	0.02	2.98 (1.18–7.47)
Age ≥ 60 years old	0.03	2.52 (1.07–5.93)
Previous therapeutic antimicrobial use	0.04	2.45 (1.06–5.65)
Liver disease	0.02	4.91 (1.25–19.29)
Bacteremia by *Klebsiella pneumoniae*	<0.001	4.59 (1.93–70.93)

(^a^) Multivariate analysis using a logistic regression model included the following variables: male sex, age ≥ 60 years old, previous therapeutic antimicrobial use, liver disease, catheter use, hospitalization in prior 6 months and bacteremia by *K. pneumoniae*

Among all the analyzed factors, male sex (*P =* 0.02), age ≥ 60 years old (*P =* 0.03), previous therapeutic antimicrobial use (*P =* 0.04), liver disease (*P* = 0.02) and bacteremia by *K. pneumoniae* (*P* = 0.0006) were associated with the presence of MDR infections. Among these factors, we can highlight the bacteremia from *K. pneumoniae* and liver disease, since patients with these characteristics had 4.6 and 4.9 times greater odds of presenting MDR infections than those who did not.

### Microbiological features

Of the 143 patients with BSI, a total of 170 bacterial isolates were recovered. *Enterobacteriaceae* were predominant (*n =* 137, 80.5%) with *Escherichia coli* (*n =* 50) and *K.pneumoniae* (*n =* 50) being most frequently isolated. Among the non-fermentative GNB isolated (*n =* 29, 16.4%), *Pseudomonas aeruginosa* (*n =* 12), *A. baumannii* (*n =* 6) and *Stenotrophomonas maltophilia* (*n* = 6) were the most frequently observed pathogens (Table 1).

**Fig. 1:**
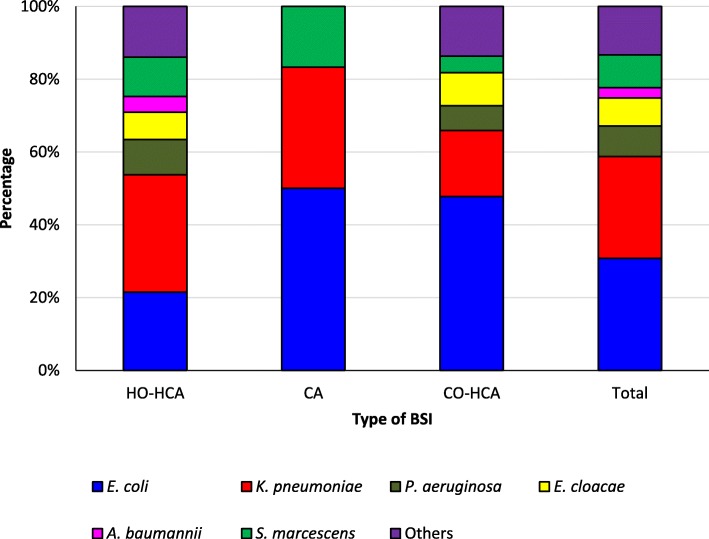
Distribution of cases of bloodstream infection (BSI) in a public-private hospital, from 2015 to 2016, based on the location of acquisition and stratified by etiology CA = community-acquired (*n =* 6), CO-HCA = community-onset, healthcare-associated (*n* = 44), HO-HCA = hospital-onset, healthcare-associated (*n* = 93). Others = *Aeromonas hydrophila* (*n* = 4), *Burkholderia cepacia* (*n* = 1), *Citrobacter koseri* (*n* = 1), *Elizabethkingia meningoseptica* (*n* = 1), *Enterobacter aerogenes* (*n* = 2), *Klebsiella oxytoca* (*n* = 1), *Moraxella osloensis* (*n* = 1), *Proteus mirabilis* (*n* = 4), *Pseudomonas putida* (*n* = 1), *Salmonella* spp. (*n* = 1), *Sphingomonas paucimobilis* (*n* = 1), and *Stenotrophomonas malthophilia* (*n* = 6). (*n* = 143)

When we stratified the pathogens based on their location of acquisition (Fig. 1), it was noted that the CA group consisted of *E. coli* (*n* = 3), *K. pneumoniae* (*n* = 2) and *S. marcescens* (*n* = 1) infections. Regarding the diversity of pathogens, the HO-HCA infections were the most heterogeneous, with *K. pneumoniae* (30/93, 32.3%) being the most frequently observed microorganism. It is noteworthy that the infections caused by *A. baumannii* occurred only in this group. For CO-HCA infections, it is also possible to see a greater diversity of pathogens, but, similar to CA, the most frequently noted microorganism was *E. coli* (21/44, 47.7%).

While stratifying the etiology of cases per months, no clusters were observed. The pathogen distribution occurred equally during the months of the study. Upon correlating the etiology and hospitalization units, *E. coli* and *K. pneumoniae* cases were present in all them; however, there was a predominance of *K. pneumoniae* in the intensive care unit (ICU) and *E. coli* in the hospital wards and emergency department.

The proportion of antimicrobial resistance among *Enterobacteriaceae* that were most frequently isolated can be seen in Table 4. *K. pneumoniae* had the highest resistance levels, including penicillins associated with β-lactamase inhibitors (52.0–62.0%), cephalosporins (50.0–52.0%), carbapenems (10.0%), fluoroquinolones (46.0%) and aminoglycosides (2.0–28.0%). It is noteworthy that two *K. pneumoniae* isolates were resistant to polymyxin. This resistance was initially detected by the VITEK 2® (bioMeriéux-France) and by E-test (bioMeriéux-France). However, this phenotype was confirmed by broth microdilution for only one isolate (minimum inhibitory concentration of 64 μg/mL).

**Table 4:** Proportions of antimicrobial resistance among *Enterobacteriaceae* that were most frequently isolated from bloodstream infections

	*Escherichia coli* (*n* = 50)		*Klebsiella pneumoniae* (*n* = 50)		*Serratia marcescens* (*n* = 50)		*Enterobacter cloacae* (*n* = 13)	
Antimicrobial drug	No. of isolates	% resistant*	No. of isolates	% resistant*	No. of isolates	% resistant*	No. of isolates	% resistant*
Amikacin	–	–	1	2.0	1	6.7	**–**	**–**
Amp-Sul	28	56.0	31	62.0	15	100	13	100
Pip-Tazo	5**	10.0**	21	52.0	1	6.7	3	23.1
Ceftriaxone	10	20.0	25	50.0	NA	NA	NA	NA
Ceftazidime	10	20.0	25	50.0	–	–	NA	NA
Cefepime	10	20.0	25	50.0	1	6.7	3	23.1
Cefuroxime	12	24.0	26	52.0	NA	NA	NA	NA
Imipenem	2	4.0	5	10.0	-^∆∆^	-^∆∆^	1	7.7
Meropenem	1	2.0	5	10.0	–	–	1	7.7
Ertapenem	1	2.0	5^+^	10.0^+^	1	6.7	2	15.4
Ciprofloxacin	15	30.0	23	46.0	1^§^	6.7^§^	5	38.5
Gentamicin	5***	10.0***	14	28.0	–	–	3	23.1
Polymyxin	–	–	1	2.0	15^§§^	100^§§^	4	30.8
Tigecycline	–	–	4^∆^	8.0^∆^	1^Ω^	6.7 ^Ω^	1 ^Ω Ω^	7.7 ^Ω Ω^

*Considered resistant or intermediary profile;** Just 1 isolate of *E. coli* was not tested against piperacillin-tazobactam; *** Just 1 isolate of *E. coli* was not tested against gentamicin; ^+^just 1 isolate of *K. pneumoniae* was not tested against ertapenem; ^∆^7 isolates of *K. pneumoniae* were not tested against tigecycline; ^∆∆^2 isolates of *S. marcescens* were not tested against imipenem; ^§^2 isolates of *S. marcescens* were not tested against ciprofloxacin; ^§§^*S. marcescens* is *intrinsically resistant* to polymyxin; ^Ω^ 1 isolate of *S. marcescens* were not tested against tigecycline; ^Ω Ω^ 3 isolates of *E. cloacae* were not tested against tigecycline; NA- Not applicable; (−) no resistant microbial isolates

Regarding the antimicrobial resistance frequency for the non-fermentative GNB (Table 5), *A. baumannii* presented very high levels of resistance when compared to *P.aeruginosa*, highlighting the resistance to penicillins associated with β-lactamase inhibitors, cephalosporins, carbapenems (66.7%) and folate pathway inhibitors (83.4%). However, all *A. baumannii* isolates were full susceptible against tigecycline.

**Table 5:** Proportions of antimicrobial resistance among non-fermentative gram-negative bacilli that were most frequently isolated from bloodstream infections

	*Pseudomonas aeruginosa* *(n = 12)*		*Acinetobacter baumannii* *(n = 6)*	
Antimicrobial drug	No. of isolates	% resistant*	No. of isolates	% resistant*
Amikacin	–	–	1	16.7
Aztreonam	4	25	1**	16.7**
Amp-Sul	NA	NA	3***	50.0***
Pip-Tazo	1	8.3	4	66.7
Ceftazidime	1	8.3	4	66.7
Cefepime	–	–	4	66.7
Imipenem	3	25.0	4	66.7
Meropenem	2	16.7	4	66.7
Ciprofloxacin	1	8.3	4	66.7
Gentamicin	–	–	2	33.3
Polymyxin	–	–	–	–
Sulfamethoxazole trimethoprim	NA	NA	5	83.4
Tobramycin	–	–	NA	NA
Tigecycline	NA	NA	-****	-****

*Considered resistant or intermediary profile;**just 1 isolate of *A. baumannii* was tested against aztreonam;*** just 1 isolate of *A. baumannii* was not tested against ampicillin-sulbactam; *** just 1 isolate of *A. baumannii* was not tested against tigecycline; NA- Not applicable; and (−) no resistant microbial isolates

Of the 170 microorganisms isolated, 158 were subjected to PCR for the detection of resistance genes, of which 84 (53.2%) were positive for at least one of the tested genes. *K. pneumoniae* was the species with the highest prevalence of resistance genes (74.0%), followed by *E. coli* (60.0%), *P. mirabilis* (50.0%), *E. cloacae* (46.2%), *A. baumannii*, (33.3%), *Aeromonas hydrophila* (25.0%) and *P. aeruginosa* (8.3%).

The *bla*_SHV_ gene was found in *n =* 42 isolates, with *bla*_TEM_ in *n =* 37; *bla*_OXA-1-like_ in *n =* 22; *bla*_CTX-M-gp1_ in *n =* 22; *bla*_KPC_ in *n =* 5; *bla*_CTX-M-gp9_ in *n =* 4; *bla*_CTX-M-gp2_, *bla*_VIM_, *bla*_GES_, *bla*_OXA-48-like_, *bla*_NDM_; and *bla*_OXA-23-like_ in *n =* 1 each. Among the isolates that caused CA infections, *n =* 3 (50%) presented resistance genes (*bla*_TEM_, *bla*_SHV_, and *bla*_CTX-M-gp9_).

The maximum number of different resistance genes found in a single isolate was *n =* 5 in a *K. pneumoniae* isolate. Isolates of the same species with similar genetic determinants of resistance were considered as having the same genetic profile. A total of 37 genetic profiles and their description can be found in Additional file 3: Table S3. The distribution of antimicrobial resistance encoding genes stratified by pathogens is shown in Additional file 4: Figure S1.

Among the 30 *E. coli* isolates and the 37 *K. pneumoniae* isolates positive for at least one resistance gene, 10 and 14 genetic resistance profiles were found, respectively. Those profiles were distributed over time (study period in months) and space (hospital units). Regarding *E. coli*, it was noted that the most frequently observed genetic profile, i.e., the one present in a greater number of isolates (*1e-bla*_TEM_, *n* = 18), was prominent during the study months. It was also observed that this profile is present in all the hospital units, with hospital wards being the units that harbored the most cases of this profile.

A similar situation was observed for the *1kp* profile of *K. pneumoniae* (*bla*_SHV_, *n* = 15). This profile was the most frequently noted among the isolates of *K. pneumoniae*, and it occurred during most months of the study period. This pattern was found in the hospital wards and semi-intensive unit, and it was predominant in the ICU setting. Unlike *E. coli*, no case of *K. pneumoniae* with resistance genes occurred in the emergency department. The ICU setting harbored a greater diversity of *K. pneumoniae* genetic profiles, albeit the profile with the highest number of resistance genes (*12kp - bla*_TEM_, *bla*_SHV_, *bla*_OXA-1-like_, *bla*_CTX-M-gp1_ and *bla*_KPC_) occurred in the semi-intensive unit.

## Discussion

Understanding the regional epidemiological and microbiological data is of great importance when handling potentially life-threatening infections such as BSI, since accuracy in predicting pathogens and the resistance profile are crucial for successful therapy [17, 31]. Surveillance studies focused on BSI are considered good choices for assessed issues related to antimicrobial resistance since the standardized clinical diagnostic criteria of those infections prevent the problem of confounding colonizing agents that are not directly associated with clinical disease, making the data more reliable [15].

Consistent with previous studies about the epidemiology of BSI that were undertaken inside and outside of Brazil, the majority of BSI occurred in male and elderly patients [12, 15, 32, 33]. The predominance of patients of the male sex who suffered this type of infection seems to be independently associated with the age group, since it was noted not only in the elderly population, but also in studies conducted in pediatric populations [16, 17, 34].

Overall, 28.7% of patients included in the present investigation had BSI due to MDR pathogens. We found that the male sex, age ≥ 60, previous antimicrobial therapy, liver disease and bacteremia caused by *K. pneumoniae* were independent factors associated with MDR infection. Studies of risk factors for MDR-GNB infections and/or colonization are difficult to compare since the study designs, populations, local-epidemiology, and definitions of resistance vary widely [35]. However, some investigations point to similar observations as those described here [36–38].

Regarding liver disease, there are reports relating this condition to a greater predisposition to bacterial infections, but there is little evidence of predisposition to the MDR phenotype [39]. We believe that over the course of the illness, patients with this chronic condition frequently need day hospital care, recurrent hospitalization or admission to intensive care units, which may increase the risk of acquiring MDR pathogens. Another reason which can explain this predisposition is the fact that those patients are often submitted to antimicrobial therapy (prophylactic and therapeutic) in order to prevent and to treat the recurrence of spontaneous bacterial peritonitis and other bacterial infections which are common in this subpopulation.

The previous use of antimicrobials is well-described in the literature as a risk factor for infection due to MDR-GNB, especially concerning cephalosporins, carbapenems and fluoroquinolones [40]. Our findings show the predominance of fluoroquinolone use in the MDR-group, although statistical significance was not reached. We hypothesized that we could not see a statistical association because our sample was small and heterogeneous (we included all the gram-negative ones, not just one species).

It is not surprising, and it is consistent with previous reports, that *K. pneumoniae* infections showed a statistical association with the MDR phenotype. This organism is notorious for its ability to accumulate and transfer resistance determinants, and it has been well-recognized as a leading causative agent of hospital-based infections over the past few decades. The association of *K. pneumoniae* with BSI caused by MDR-GNB has already been reported in Europe, Asia, and South America [41–44].

Most studies on the epidemiology of BSIs have focused on hospital-onset infections alone. Our study examines community-onset and hospital-onset healthcare-associated infections (CO-HCA and HO-HCA) and community-acquired infections (CA), allowing us to elucidate the differences between them regarding the resistance profile and causative microorganisms. However, we had a low proportion of community-acquired cases (*n* = 6, 4.2%), which may have been explained by the profile of the São Rafael Hospital, since most of the patients attending this institution have chronic healthcare conditions. These small numbers of cases have limited our ability to make inferences about the epidemiology of these infections.

Nevertheless, it was possible to note the HO-HCA infections harbored a greater number of MDR cases (*n* = 29, 70.7%) compared to the CO-HCA infections (*n* = 11, 26.8%) and CA infections (*n* = 1, 2.4%), which has biological plausibility since hospitals are widely recognized as environments that multiply and disseminates MDR microorganisms [45]. Regarding the etiology of CA and CO-HCA infections, the most frequently noted pathogen was *E. coli*. The HO-HCA infections were mostly made up of *K. pneumoniae* cases, and *A. baumannii* was found only in this type of infection, which is consistent with previous reports [15, 17, 44].

The antibiotic resistance proportions found in our study are worrisome, especially regarding *K. pneumoniae*, which was the *Enterobacteriaceae* with the highest resistance level, at 52.0% to cephalosporins and 10.0% to carbapenems, and *A.baumannii*, which presented 66.7% to cephalosporins and carbapenems and 83.4% to sulfametoxazole-trimethoprim. Our data is consistent with those described by Marra et al. [15] that evaluated the epidemiology of nosocomial BSI from a prospective nationwide surveillance study. This data came from hospitals geographically dispersed throughout the five different regions of Brazil, suggesting the important external validity of our study.

In contrast to the Brazilian scientific literature data on the emergence of β-lactamases [41, 45], which shows that cefotaximases are the most detected β-lactamase, especially CTX-M-gp2, the most frequent resistance determinants in our evaluation were *bla*_SHV_ (*n* = 42) and *bla*_TEM_ (*n* = 37), followed by *bla*_OXA-1-like_ (*n* = 22), *bla*_CTX-M-gp1_ (*n* = 22), *bla*_CTX-M-gp9_ (*n* = 4) and *bla*_CTX-M-gp2_ (*n* = 1). However, *K.pneumoniae* was the microbial species with the highest prevalence of resistance genes (74.0%), which is concordant with previous findings [38]. It is worth mentioning that, albeit we have few isolates from CA infections, half of them presented β-lactamases genes (*bla*_TEM_, *bla*_SHV_, and *bla*_CTX-M-gp9_), demonstrating the spread of these genes in off-hospital environments.

Concerning the carbapenemase genetic determinants, the *bla*_KPC_ was the most frequently noted, followed by *bla*_OXA-23-like_, while *bla*_VIM_, *bla*_GES_, *bla*_OXA-48-like_, and *bla*_NDM_ were the least frequently noted. In Latin America, Brazil stands out as one of the countries in which carbapenemases are most frequently reported. The *bla*_KPC_ gene is widely distributed throughout the country and its occurrence was already described in *K. pneumoniae*, *Enterobacte*r spp., *E. coli*, *S. marcescens*, and *K. oxytoca* clinical isolates [46]. Recently, Tavares et al. [47] reported the presence of the KPC-2 gene in the state of Bahia for the first time, in *E. coli* isolates. In our study 4/5 (80%) of KPC producers were *K. pneumoniae* isolates, whereas 20% were *E.coli*.

The first clinical report of *bla*_NDM_ in northeast Brazil was in a public hospital in Salvador, recently described by Barberino et al. [48]. In our investigation, the presence of the *bla*_NDM_ gene was verified in an *E. coli* isolate; however this strain did not express phenotypic resistance to carbapenems.

The present study has the major advantage of being prospective, with a clear and consensual definition of infection. However, it also has significant limitations that should be acknowledged. Cases in which bacterial isolates were not stored were not included in this investigation. Furthermore, even though, this evaluation corresponds to the beginning of a surveillance network to track antimicrobial resistance in hospitals in the city of Salvador, our analyses reflect the profile of only one institution. Therefore, they cannot be extrapolated to the general population or applied to the situations of other hospitals. Nevertheless, the results presented here constitute the first step for future multicenter studies focusing on avoid the dissemination of the resistance genes in the region of Salvador.

## Conclusions

In summary, we found that the factors associated with MDR bacteremia were liver disease, male sex, age ≥ 60, previous therapeutic antimicrobial use and bacteremia caused by *K. pneumoniae*. Regarding resistance determinants, SHV, TEM, OXA-1-like and CTX-M-gp1 were the predominant enzymatic variants, whereas CTX-M-gp9, CTX-M-gp2, KPC, VIM, GES, OXA-48-like, NDM and OXA-23-like can be characterized as emerging enzymes.

## Additional files

Additional file 1: **Table S1.** Primers and thermocycling conditions used in polymerase chain reactions (PCR). Primers and thermocycling conditions used in polymerase chain reactions (PCR) (DOCX 21 kb)

Additional file 2: **Table S2.** Reference strains used as controls in polymerase chain reactions (PCR). (DOCX 14 kb)

Additional file 3: **Table S3.** Genetic profile of resistance among eighty-for isolates that were positive for ESBL and carbapenemases genes according PCR results. (**a)**-*Acinetobacter baumannii*, (**ec**)- *Enterobacter cloacae*, (**e**)*- Escherichia coli*, (**kp**)- *Klebsiella pneumoniae*, (**pa**)- *Pseudomonas aeruginosa*, (**pm**)-*Proteus mirabilis*, (**em**) *Elizabethkingia meningoseptica*, (pp) *Pseudomonas putida*, (sp) *Sphingomomas paucimobilis*, (ah) *Aeromonas hydrophila*. (DOCX 21 kb)

Additional file 4: **Figure S1.** Antimicrobial resistance encoding genes stratified by pathogens. *bla*_TEM_ (*n =* 37), *bla*_SHV_ (*n* = 42), *bla*_OXA-1-like_ (*n* = 22), *bla*_CTX-M-1_(*n* = 22), *bla*_CTX-M-2_ (*n* = 1), *bla*_CTX-M-9_ (*n* = 4), *bla*_GES_ (*n* = 1), *bla*_OXA-48-like_ (*n* = 1),*bla*_KPC_ (*n* = 5),*bla*_VIM_ (*n* = 1), *bla*_NDM_ (*n* = 1), *bla*_OXA-23-like_ (*n* = 4). Others = *Aeromonas hydrophila* (*bla*_SHV_ positive *n* = 1), *Elizabethkingia meningoseptica* (*bla*_CTX-M-1_ positive *n* = 1), *Proteus mirabilis* (*bla*_TEM_ positive *n* = 1; *bla*_OXA-23-like_*n* = 3), *Pseudomonas putida* (*bla*_VIM_ positive, *n* = 1), *Sphingomonas paucimobilis* (*bla*_GES_ positive, *n* = 1). (*n =* 84). (PPTX 63 kb)

## Data Availability

The datasets used in the current study are not publicly available to maintain the privacy and confidentiality of the participants but are available from the corresponding author on reasonable request.

## Abbreviations

°C: Degree Celsius
*bla*: β-lactamase
BSI: Bloodstream Infection
CA: Community-acquired bloodstream infection
CDC: Centers of Disease Control and Prevention
CLSI: Clinical and Laboratory Standards Institute
CO-HCA: Community-onset, healthcare-associate bloodstream infection
CTX-M-gp1: Cefotaximases group 1
CTX-M-gp2: Cefotaximases group 2
CTX-M-gp9: Cefotaximases group 9
ESβL: Extended Spectrum β-lactamase
Fiocruz: Fundação Oswaldo Cruz
HO-HCA: Hospital-onset, healthcare-associated bloodstream infection
HSR: Hospital São Rafael
M: Molar
MALDI-TOF/MS: Matrix-Assisted Laser Desorption Ionization-Time of Flight/ Mass Spectrometry
MDR: multidrug resistance
mg: Milligram
mL: Milliliter
mM: Millimolar
pH: Hydrogen potential
μg: Microgram
μL: Microliter
SIRS: Systemic Inflammatory Response Syndrome
SUS: Sistema Único de Saúde
TBE: Tris-Borate-EDTA
ρmol: picomol
