# Supplementary material for: Bloodstream infections caused by multidrug-resistant gram-negative bacteria: epidemiological, clinical and microbiological features
Source: BMC Infect Dis. 2019 Jul 11;19:609. doi: 10.1186/s12879-019-4265-z (PMC6624930; doi:10.1186/s12879-019-4265-z)
Supplement: Supplementary file 1 — Table S1. Primers and thermocycling conditions used in polymerase chain reactions (PCR). Primers and thermocycling conditions used in polymerase chain reactions (PCR) (DOCX 21 kb) [file 12879_2019_4265_MOESM1_ESM.docx]

**Supplementary Table 1** – Primers and thermocycling conditions used in polymerase chain reactions (PCR).

| **PCR** | **Primer name** | **[pmol/μl]** | **Primer sequence (5’-3’)** | **Gene** | **Aplicom length** | **Thermocycling conditions** |
| --- | --- | --- | --- | --- | --- | --- |
| Multiplex I – *^bla^*TEM, *^bla^*SHV, *^bla^*OXA | MultiTSO-T_for | 0,4 | CAT TTC CGT GTC GCC CTT ATT C | TEM-1 e TEM-2 variants | 800 | Desnaturation:94°C -10 min  Desnaturation: 94°C - 40s  Annealing: 60°C - 40s  Extension: 72°C - 1min.  30 cycles  Final extension:72°C -7min. |
|  | MultiTSO-T_rev | 0,4 | CGT TCA TCC ATA GTT GCC TGA C |  |  |  |
|  | MultiTSO-S_for | 0,4 | AGC CGC TTG AGC AAA TTA AAC | SHV, including SHV-1 variant | 713 |  |
|  | MultiTSO-S_rev | 0,4 | ATC CCG CAG ATA AAT CAC CAC |  |  |  |
|  | MultiTSO-O_for | 0,4 | GGC ACC GAT TCA ACT TTCA AG | OXA-1, OXA-4 e OXA-30 variants | 564 |  |
|  | MultiTSO-O_rev | 0,4 | GAC CCC AAG TTT CCT GTA AGT G |  |  |  |
| Multiplex II –*^bla^*CTX-M | Ctx-mmulti- grp1 f | 0,4 | TTA GGA ART GTG CCG CTG YA | CTX-M-Group 1 CTX-M-1, CTX-M-3 e CTX-M-15 | 688 |  |
|  | Ctx-mmulti- grp1 r | 0,2 | CGA TAT CGT TGG TGG TRC CAT |  |  |  |
|  | Ctx-mmulti- grp2 r | 0,2 | CGT TAA CGG CAC GAT GAC | CTX-M Group 2 - including CTX-M-2 | 404 |  |
|  | Ctx-mmulti- grp2 f | 0,2 | CGA TAT CGT TGG TGG TRC CAT* |  |  |  |
|  | Ctx-mmulti- grp9 f | 0,4 | TCA AGC TGC CAT CGG T | CTX-M Group 9 | 561 |  |
|  | Ctx-mmulti- grp9 r | 0,4 | TGA TTC TCG CCG CTG AAG |  |  |  |
| Multiplex III –*^bla^*GES, *^bla^*OXA-48-like | MultiGES_for | 0,4 | AGC GGC TAG ACC GGA AAG | GES -1 a GES-9 and GES-11variants | 399 |  |
|  | MultiGES_rev | 0,4 | TTT GTC CGT GCT CAG GAT |  |  |  |
|  | MultiOXA-8_for | 0,4 | GCT TGA TCG CCC TCG ATT | OXA-48-like | 281 |  |
|  | MultiOXA-8_rev | 0,4 | TTCGGCTTGACTCGGCTGA |  |  |  |
| Multiplex VI –*^bla^*KPC, *^bla^*VIM | MultiVIM_for | 0,5 | GATGGTGTTTGGTCGCATA | VIM, including VIM-1 e VIM-2 variants | 390 |  |
|  | MultiVIM_rev | 0,5 | CGAATGCGCAGCACCAG |  |  |  |
|  | MultiKPC_for | 0,2 | CATTCAAGGGCTTTCTTGCTGC | KPC-1 to KPC-4 variants | 538 |  |
|  | MulbtiKPC_rev | 0,2 | ACG ACG GCA TAG TCA TTT GC |  |  |  |

**Supplementary Table 1 (continuation)** – Primers and thermocycling conditions used in polymerase chain reactions (PCR).

| **PCR** | **Primer name** | **[pmol/μl]** | **Primer sequence (5’-3’)** | **Gene** | **Aplicom length** | **Thermocycling conditions** |
| --- | --- | --- | --- | --- | --- | --- |
| Simplex I – *^bla^*IMP | IMP-F | 0,4 | GGA ATA GAG TGG CTT AAY TCT C | IMP variants | 233 | Desnaturation:94°C -10 min  Desnaturation: 95°C – 1min  Annealing: 57,5°C – 1min  Extension: 72°C - 1min.  25 cycles  Final extension:72°C -7min. |
|  | IMP-R2 | 0,4 | GGT TTA AYA AAA CAA CCA CC |  |  |  |
| Simplex II – *^bla^*NDM | NDM_MP-F | 0,24 | TCCTTGATCAGGCAGCCACC | NDM variants | 591 |  |
|  | NDM_MP-R | 0,24 | CGCATTAGCCGCTGCATTGA |  |  |  |
| Simplex III – *^bla^*OXA-23-like | OXA_23-F | 0,4 | GAT CGG ATT GGA GAA CCA GA | OXA-23-like | 501 |  |
|  | OXA_23-R | 0,4 | ATTTCTGACCGCATTTCCAT |  |  |  |
