# Supplementary material for: Bloodstream infections caused by multidrug-resistant gram-negative bacteria: epidemiological, clinical and microbiological features
Source: BMC Infect Dis. 2019 Jul 11;19:609. doi: 10.1186/s12879-019-4265-z (PMC6624930; doi:10.1186/s12879-019-4265-z)
Supplement: Supplementary file 2 — Table S2. Reference strains used as controls in polymerase chain reactions (PCR). (DOCX 14 kb) [file 12879_2019_4265_MOESM2_ESM.docx]

**Supplementary Table 2**- Reference strains used as controls in polymerase chain reactions (PCR).

| **Reference strains** |
| --- |
| *Eescherichia coli* 300 (*bla*_TEM -1_) |
| *Klebsiella pneumoniae* ATCC 700603 (*bla*_SHV-18_) |
| *Pseudomonasaeruginosa* (*bla*_OXA-161_ e *bla*_CTX-M-9_) |
| *Klebsiella pneumoniae kp13* (*bla_KPC-2_*) |
| *Escherichia coli* 455 (*bla*_CTX-M-15_) |
| *Escherichia coli* 300 (*bla*_CTX-M-2_) |
| *Raoultella ornithinolytica (bla*_OXA-48-like_*)* |
| *Pseudomonas fluorescens* CCBH 11805 (*bla*_VIM_) |
| *Acinetobacter baumannii* **(***bla*_OXA-27_) |
| *Enterobacter cloacae* CCB410882 (*bla*_NDM_) |
| *Klebsiella pneumoniae (bla_IMP_)* |
