# Supplementary material for: Bloodstream infections caused by multidrug-resistant gram-negative bacteria: epidemiological, clinical and microbiological features
Source: BMC Infect Dis. 2019 Jul 11;19:609. doi: 10.1186/s12879-019-4265-z (PMC6624930; doi:10.1186/s12879-019-4265-z)
Supplement: Supplementary file 3 — Table S3. Genetic profile of resistance among eighty-for isolates that were positive for ESBL and carbapenemases genes according PCR results. (a)-Acinetobacter baumannii, (ec)- Enterobacter cloacae, (e)- Escherichia coli, (kp)- Klebsiella pneumoniae, (pa)- Pseudomonas aeruginosa, (pm)-Proteus mirabilis, (em) Elizabethkingia meningoseptica, (pp) Pseudomonas putida, (sp) Sphingomomas paucimobilis, (ah) Aeromonas hydrophila. (DOCX 21 kb) [file 12879_2019_4265_MOESM3_ESM.docx]

**Supplementary Table 3 –** Genetic profile of resistance among eighty-for isolates that were positive for ESBL and carbapenemases genes according PCR results. (**a)**-*Acinetobacter baumannii*, (**ec**)- *Enterobacter cloacae*, (**e**)*- Escherichia coli*, (**kp**)- *Klebsiella pneumoniae*, (**pa**)- *Pseudomonas aeruginosa*, (**pm**)-*Proteus mirabilis*, (**em**) *Elizabethkingia meningoseptica*, (pp) *Pseudomonas putida*, (sp) *Sphingomomas paucimobilis*, (ah) *Aeromonas hydrophila*.

|  | **Penicillins**  **Cephalosporins**  **Monobactans** | | | | | | **Carbapenems** | | | | | | | ***N*** |
| --- | --- | --- | --- | --- | --- | --- | --- | --- | --- | --- | --- | --- | --- | --- |
| **Genetic profile** | *^bla^*TEM | *^bla^*SHV | *^bla^*OXA-1-like | *^bla^*CTX-M-1 | *^bla^*CTX-M-2 | *^bla^*CTX-M-9 | *^bla^*GES | *^bla^*OXA-48-like | *^bla^*KPC | *^bla^*VIM | *^bla^*IMP | *^bla^*NDM | *^bla^*OXA-23-like |  |
| 1pa |  |  |  |  |  |  |  |  |  |  |  |  |  | *1* |
| 1em |  |  |  |  |  |  |  |  |  |  |  |  |  | *1* |
| 1pp |  |  |  |  |  |  |  |  |  |  |  |  |  | *1* |
| 1sp |  |  |  |  |  |  |  |  |  |  |  |  |  | *1* |
| 1ah |  |  |  |  |  |  |  |  |  |  |  |  |  | *1* |
| 1ª |  |  |  |  |  |  |  |  |  |  |  |  |  | *1* |
| 2ª |  |  |  |  |  |  |  |  |  |  |  |  |  | *1* |
| 1pm |  |  |  |  |  |  |  |  |  |  |  |  |  | *3* |
| 2pm |  |  |  |  |  |  |  |  |  |  |  |  |  | *1* |
| 1ec |  |  |  |  |  |  |  |  |  |  |  |  |  | *4* |
| 2ec |  |  |  |  |  |  |  |  |  |  |  |  |  | *2* |
| 3ec |  |  |  |  |  |  |  |  |  |  |  |  |  | *1* |
| 1e |  |  |  |  |  |  |  |  |  |  |  |  |  | *18* |
| 2e |  |  |  |  |  |  |  |  |  |  |  |  |  | *2* |
| 3e |  |  |  |  |  |  |  |  |  |  |  |  |  | *2* |
| 4e |  |  |  |  |  |  |  |  |  |  |  |  |  | *2* |
| 5e |  |  |  |  |  |  |  |  |  |  |  |  |  | *1* |
| 6e |  |  |  |  |  |  |  |  |  |  |  |  |  | *1* |
| 7e |  |  |  |  |  |  |  |  |  |  |  |  |  | *1* |
| 8e |  |  |  |  |  |  |  |  |  |  |  |  |  | *1* |
| 9e |  |  |  |  |  |  |  |  |  |  |  |  |  | *1* |
| 10e |  |  |  |  |  |  |  |  |  |  |  |  |  | *1* |
| 1kp |  |  |  |  |  |  |  |  |  |  |  |  |  | *15* |
| 2kp |  |  |  |  |  |  |  |  |  |  |  |  |  | *5* |
| 3kp |  |  |  |  |  |  |  |  |  |  |  |  |  | *2* |
| 4kp | 1 |  |  |  |  |  |  |  |  |  |  |  |  | *2* |
| 5kp |  |  |  |  |  |  |  |  |  |  |  |  |  | *2* |
| 6kp |  |  |  |  |  |  |  |  |  |  |  |  |  | *2* |
| 7kp |  |  |  |  |  |  |  |  |  |  |  |  |  | *1* |
| 8kp |  |  |  |  |  |  |  |  |  |  |  |  |  | *1* |
| 9kp |  |  |  |  |  |  |  |  |  |  |  |  |  | *1* |
| 10kp |  |  |  |  |  |  |  |  |  |  |  |  |  | *1* |
| 11kp |  |  |  |  |  |  |  |  |  |  |  |  |  | *1* |
| 12kp |  |  |  |  |  |  |  |  |  |  |  |  |  | *1* |
| 13kp |  |  |  |  |  |  |  |  |  |  |  |  |  | *1* |
| 14kp |  |  |  |  |  |  |  |  |  |  |  |  |  | *1* |
