# Supplementary material for: Bloodstream infections caused by multidrug-resistant gram-negative bacteria: epidemiological, clinical and microbiological features
Source: BMC Infect Dis. 2019 Jul 11;19:609. doi: 10.1186/s12879-019-4265-z (PMC6624930; doi:10.1186/s12879-019-4265-z)
Supplement: Supplementary file 4 — Figure S1. Antimicrobial resistance encoding genes stratified by pathogens. blaTEM (n = 37), blaSHV (n = 42), blaOXA-1-like (n = 22), blaCTX-M-1(n = 22), blaCTX-M-2 (n = 1), blaCTX-M-9 (n = 4), blaGES (n = 1), blaOXA-48-like (n = 1),blaKPC (n = 5),blaVIM (n = 1), blaNDM (n = 1), blaOXA-23-like (n = 4). Others = Aeromonas hydrophila (blaSHV positive n = 1), Elizabethkingia meningoseptica (blaCTX-M-1 positive n = 1), Proteus mirabilis (blaTEM positive n = 1; blaOXA-23-liken = 3), Pseudomonas putida (blaVIM positive, n = 1), Sphingomonas paucimobilis (blaGES positive, n = 1). (n = 84). (PPTX 63 kb) [file 12879_2019_4265_MOESM4_ESM.pptx]

## Slide 1
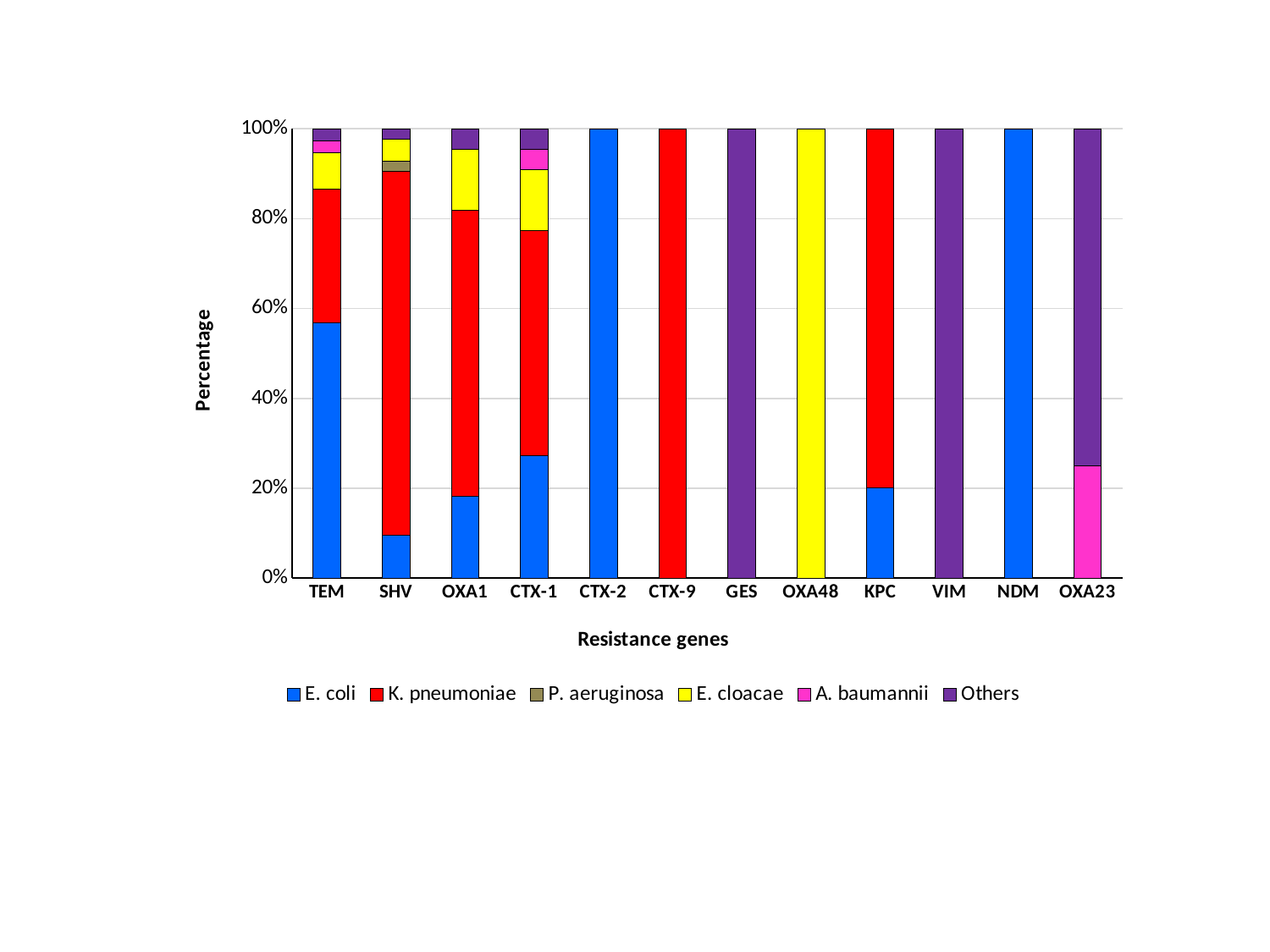

### Chart
| Category | E. coli | K. pneumoniae | P. aeruginosa | E. cloacae | A. baumannii | Others |
|---|---|---|---|---|---|---|
| TEM | 21.0 | 11.0 | 0.0 | 3.0 | 1.0 | 1.0 |
| SHV | 4.0 | 34.0 | 1.0 | 2.0 | 0.0 | 1.0 |
| OXA1 | 4.0 | 14.0 | 0.0 | 3.0 | 0.0 | 1.0 |
| CTX-1 | 6.0 | 11.0 | 0.0 | 3.0 | 1.0 | 1.0 |
| CTX-2 | 1.0 | 0.0 | 0.0 | 0.0 | 0.0 | 0.0 |
| CTX-9 | 0.0 | 4.0 | 0.0 | 0.0 | 0.0 | 0.0 |
| GES | 0.0 | 0.0 | 0.0 | 0.0 | 0.0 | 1.0 |
| OXA48 | 0.0 | 0.0 | 0.0 | 1.0 | 0.0 | 0.0 |
| KPC | 1.0 | 4.0 | 0.0 | 0.0 | 0.0 | 0.0 |
| VIM | 0.0 | 0.0 | 0.0 | 0.0 | 0.0 | 1.0 |
| NDM | 1.0 | 0.0 | 0.0 | 0.0 | 0.0 | 0.0 |
| OXA23 | 0.0 | 0.0 | 0.0 | 0.0 | 1.0 | 3.0 |
